# Supplementary figures and images for: Long lived liver-resident memory T cells of biased specificities for abundant sporozoite antigens drive malaria protection by radiation-attenuated sporozoite vaccination
Source: PLoS Pathog. 2025 May 27;21(5):e1012731. doi: 10.1371/journal.ppat.1012731 (PMC12143544; doi:10.1371/journal.ppat.1012731)

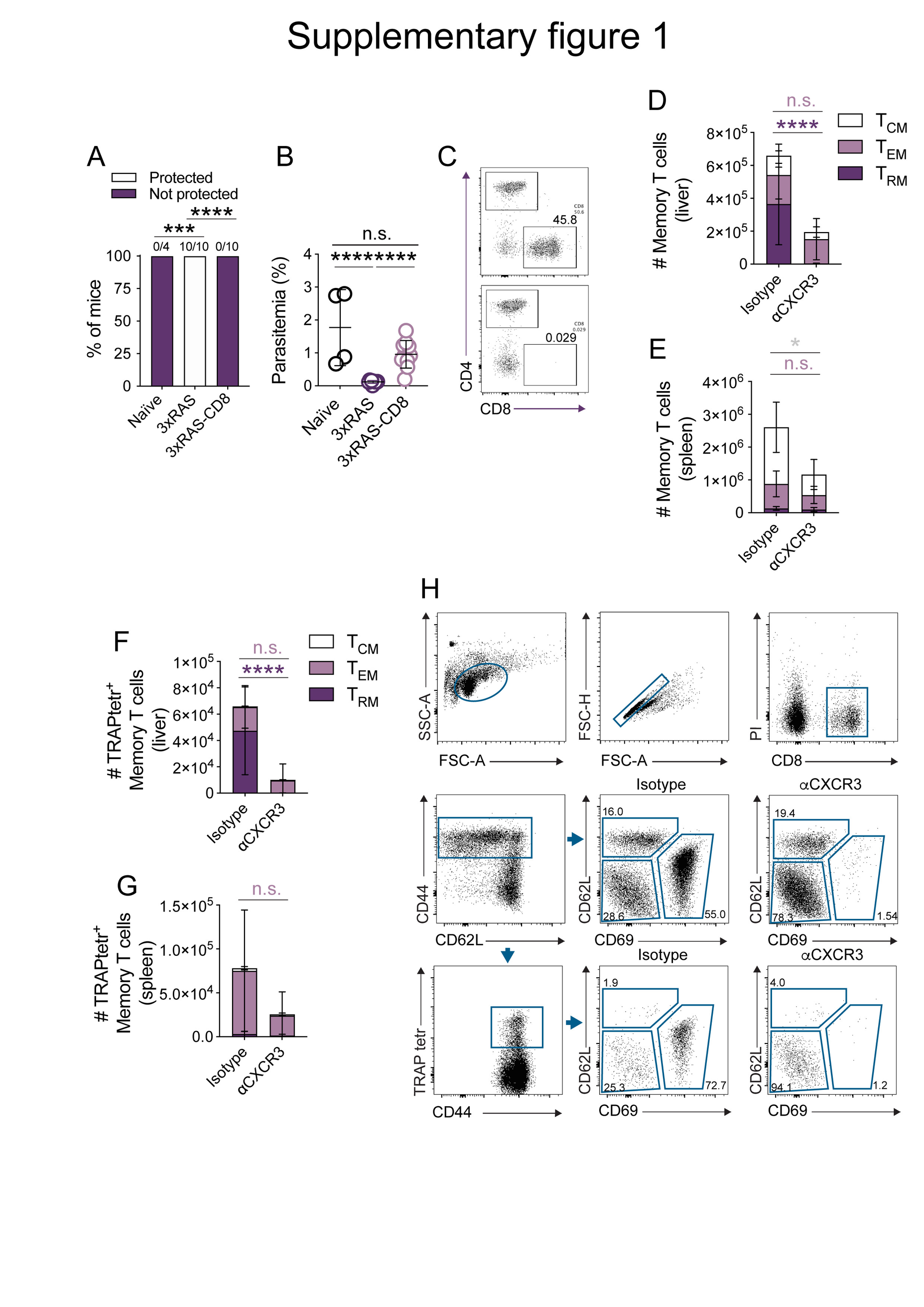

Supplement: S1 Fig — Mice vaccinated thrice with 10,000 RAS, one week apart, were treated with anti-CD8 antibodies on day 27 after the third RAS immunisation and were challenged with 200 live P. berghei sporozoites on day 30. A. Rates of sterile protection. Numbers above columns denote numbers of protected mice/ total numbers of mice per group. B. Parasitemia at day 7 post-challenge. C. Mice were bled on day 29 after the third RAS vaccination (i.e., 2 days after αCD8 treatment) and percentages of CD4+ and CD8+ T cells (showed as numbers above the CD8+ T cell gate) were measured in the blood using flow cytometry to verify CD8+ T cell depletion. An example of CD8+ T cell percentages in an untreated (top) and a treated (bottom) mouse are shown. One experiment was performed. Comparisons of sterile protection rates were done using Fisher’s exact tests. Parasitemia data were log-transformed and compared using one-way ANOVA and Tukey’s multiple comparisons tests. D-H. Related to Fig 1C and 1D. Distribution of memory T cell populations in the liver (D, F) and the spleen (E, G) in mice treated with αCXCR3 or isotype control mAb. D and E show total memory CD8+ T cells, whereas F and G show PbTRAP130–138 tetramer-specific memory CD8+ T cells. Cell numbers were log-transformed and compared using unpaired t-tests. Dark purple, pale purple and grey stats over the columns denote comparisons of TRM, TEM and TCM numbers respectively. H. Representative gating strategy of liver cells, including lymphocytes, single cells, live CD8+ T cells (CD8+ Propidium Iodide [PI]-), memory T cells (CD44high), TRAP-tetramer+ cells (gated from memory T cells) and total (middle row) or TRAP-specific (bottom row) memory T cell subsets TCM CD62L+ CD69-, TEM CD62L- CD69- and TRM CD62L- CD69+. Panels titled “Isotype” and “αCXCR3” show total (middle row) or TRAP-specific (bottom row) memory T cells in the livers of a representative, isotype-treated and αCXCR3-treated mouse respectively. Numbers beside gates represent percentag [file ppat.1012731.s001.tif]

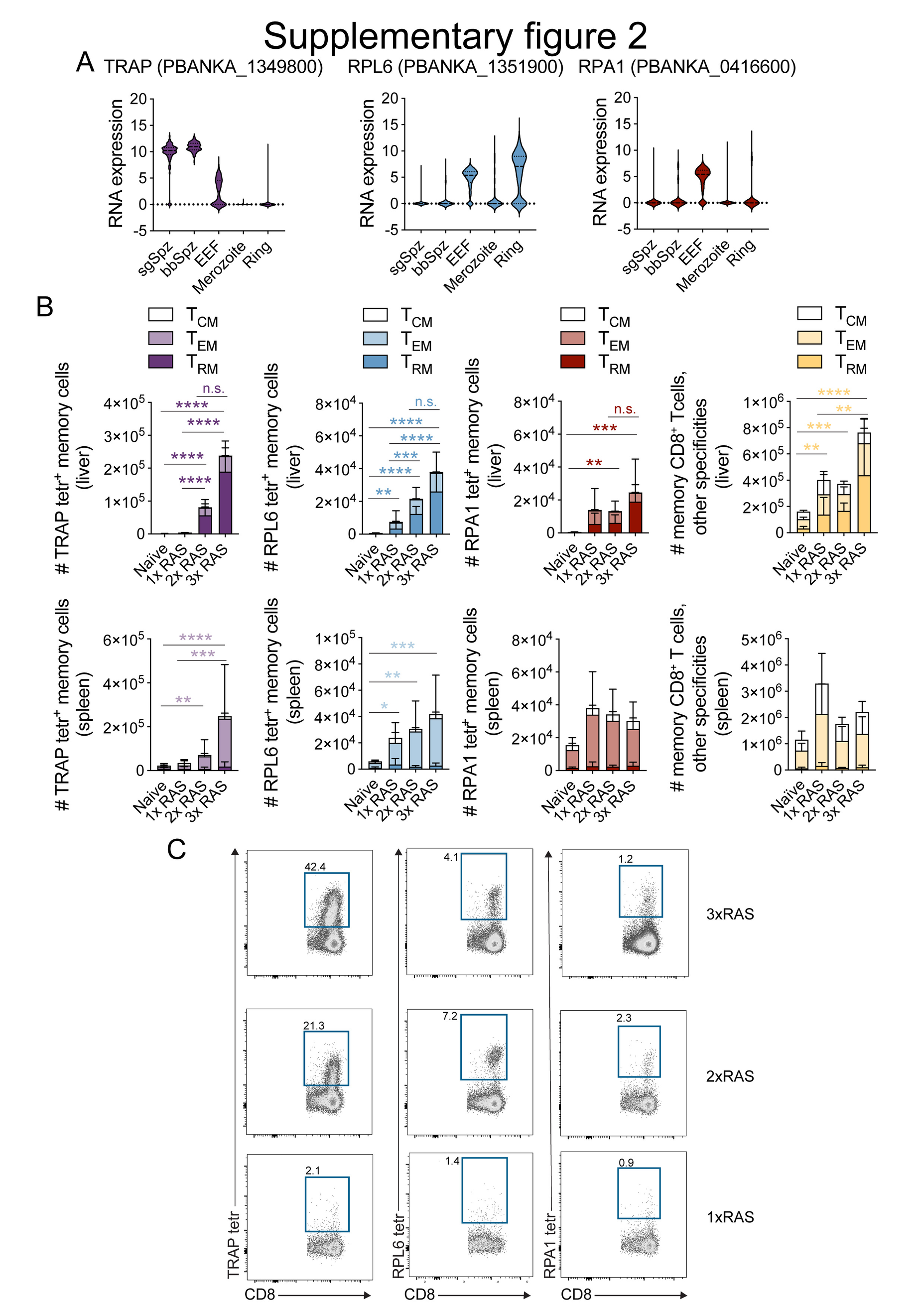

Supplement: S2 Fig — A. Expression of TRAP, RPL6 and RPA1 proteins in salivary gland sporozoites (sgSpz), injected sporozoites (bbSpz), exo-erythrocytic forms (EEF), merozoite and ring forms of P. berghei, as per the Malaria Cell Atlas [33]. B. Related to Fig 2A-C. Detailed distribution of memory CD8+ T cells of known (tetramer-positive, as indicated) or unknown (tetramer-negative) specificities in the spleen and the liver. Data were compared using one-way ANOVA and Tukey’s multiple comparisons test. The statistical analysis performed on liver data (dark asterisks) compared numbers of TRM cells, and that in spleen data (pale asterisks) compared numbers of TEM cells. C. Representative FACS plots of tetramer+ TRM cells in the liver. (TIF) [file ppat.1012731.s002.tif]

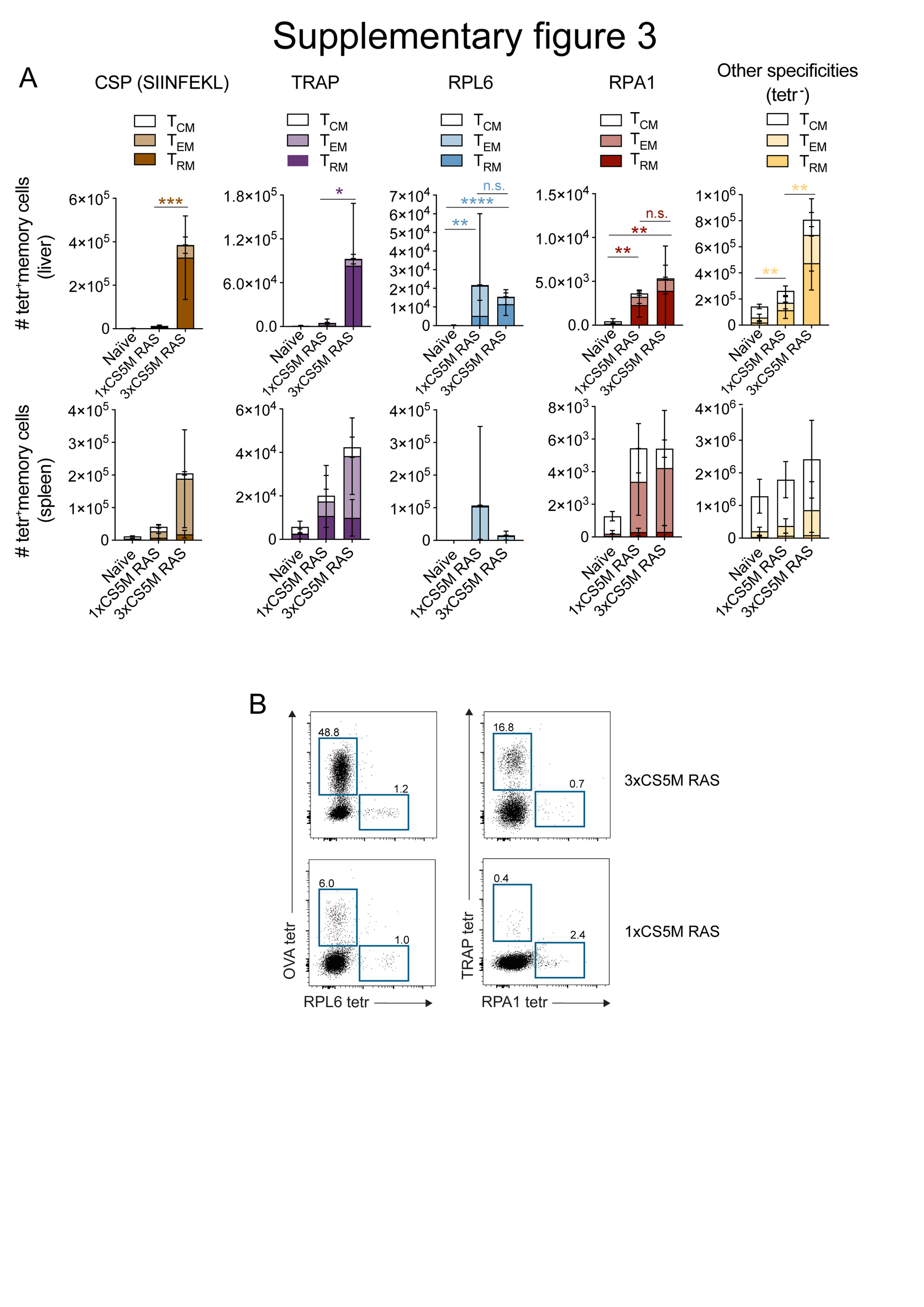

Supplement: S3 Fig — Detailed distribution of memory CD8+ T cells of known specificities in the spleen in mice vaccinated with 1x CS5M RAS or 3x CS5M RAS. A. Data were compared using one-way ANOVA and Tukey’s multiple comparisons test. The statistical analysis performed on liver data (dark asterisks) compared numbers of TRM cells. B. Representative FACS plots of tetramer+ memory CD8+ T cells in the liver (1x vs 3xCS5M RAS vaccinated mice). (TIF) [file ppat.1012731.s003.tif]

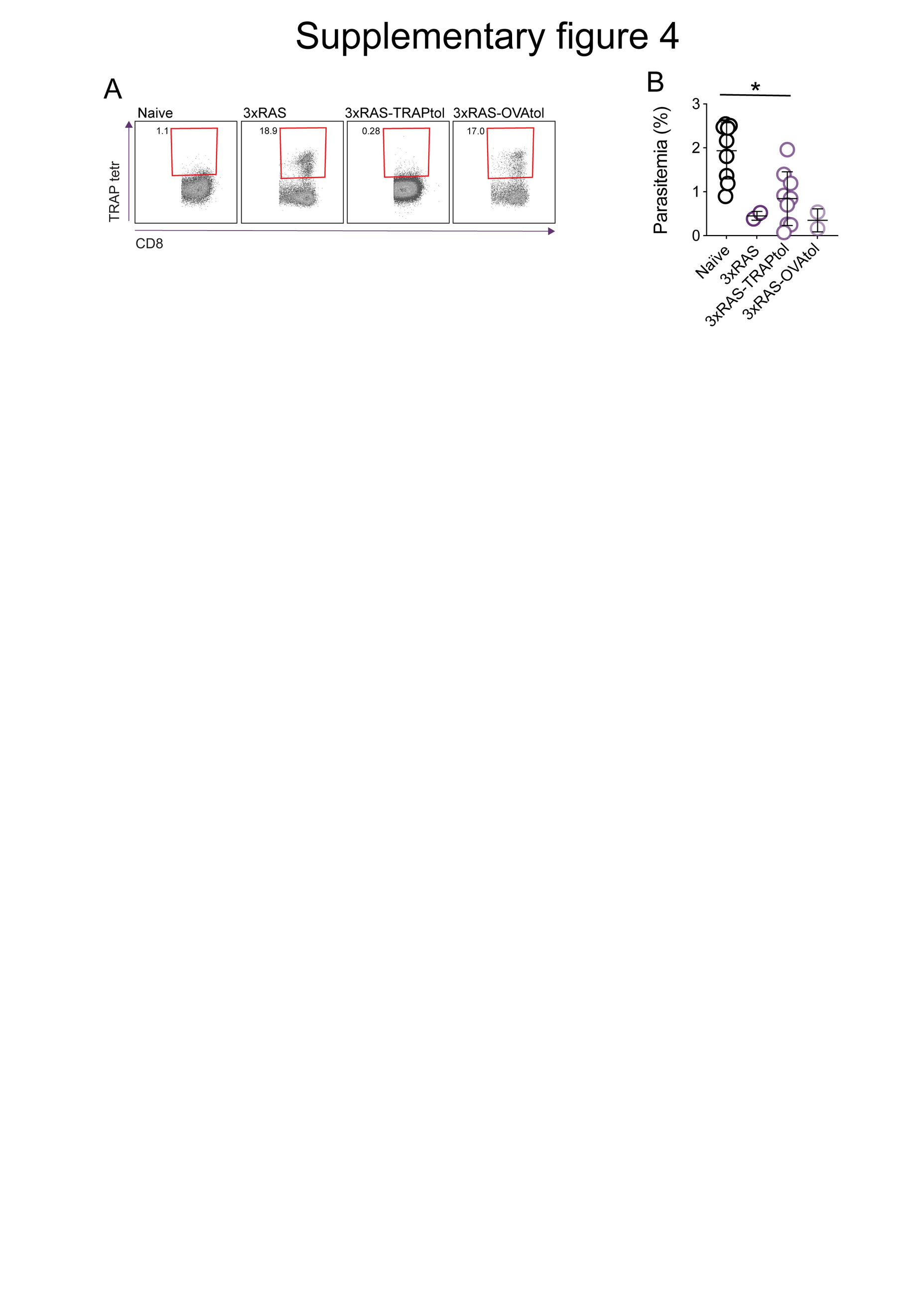

Supplement: S4 Fig — A. Related to Fig 4B-D. Representative flow cytometry charts showing depletion efficacy of TRAP specific cells. B. Related to Fig 4C. Comparison of the parasitemias of those mice that were not sterilely protected. (TIF) [file ppat.1012731.s004.tif]

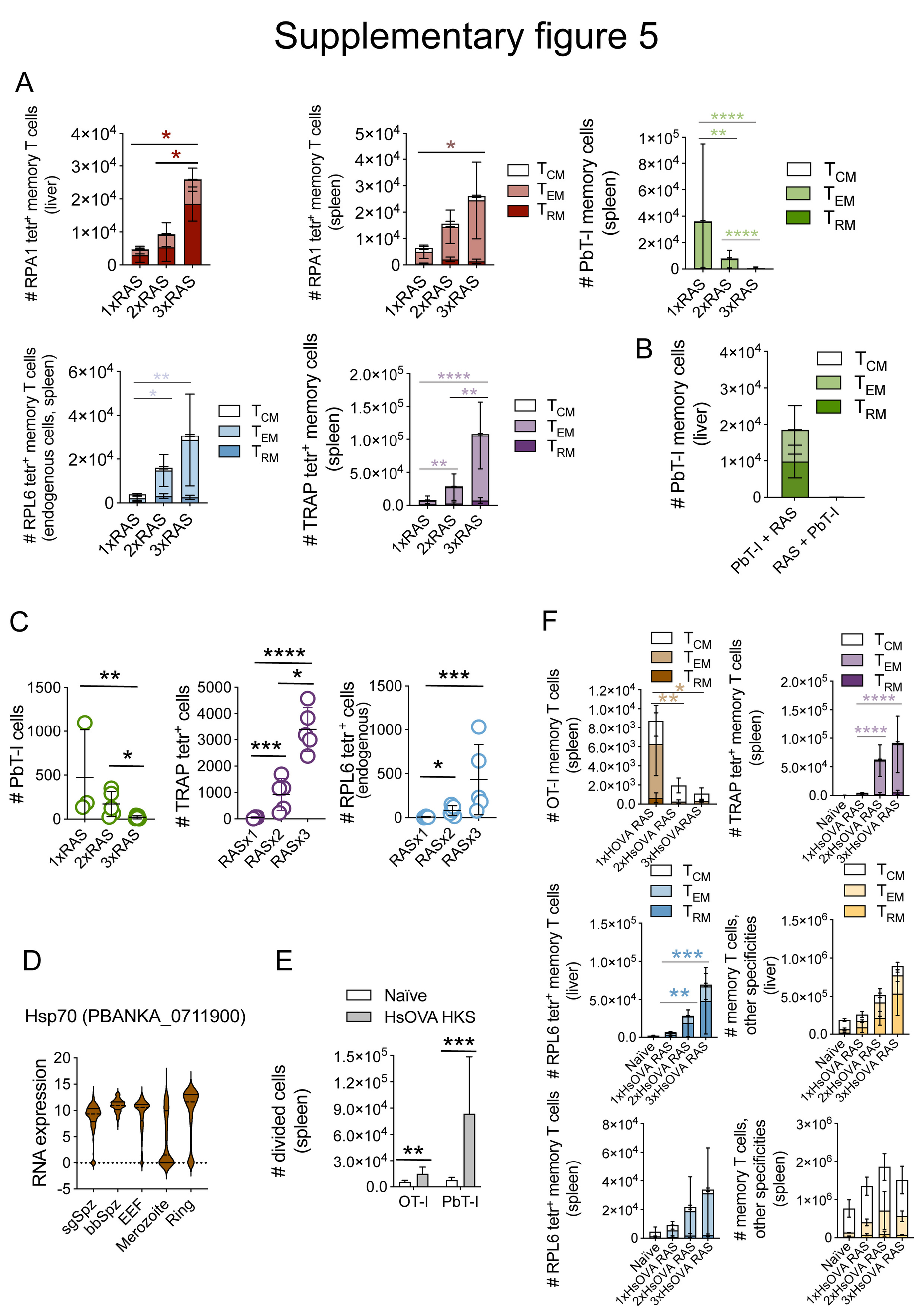

Supplement: S5 Fig — A. Related to Fig 5D-G. Distribution of PbT-I and endogenous, TRAP-, RPL6- and RPA1-specific memory T cells in the spleen and the liver as indicated. Numbers of TEM cells were statistically compared in the spleen (pale green or purple asterisks), and numbers of TRM cells were compared in the liver (dark asterisks). PbT-I and TRAP specific cell data were pooled from two independent experiments, and RPL6 and F4 cell data come from one experiment. Data were log-transformed and compared using one-way ANOVA and Tukey’s multiple comparisons test. B. Memory PbT-I cells in the liver on day 30 after transfer of 50,000 naïve PbT-I cells into mice that had been vaccinated with 10,000 RAS 6 days earlier (RAS+PbT-I), or one day later (PbT-I+ RAS). Data were pooled from two independent experiments. C. Number of PbT-I cells, TRAP- and RPL6-specific CD8+ T cells in the blood on day 7 after the last RAS vaccination. Data come from one experiment and were log-transformed and compared using one-way ANOVA and Tukey’s multiple comparisons test. D-F. Related to Fig 5H and 5I. D. Expression of Hsp70 across different life stages of the parasite, as per the Malaria Cell Atlas [33]. sgSpz, salivary gland sporozoites; bbSp, injected sporozoites; EEF, exo-erythrocytic forms. E. OT-I and PbT-I cell expansion after HsOVA HKS injection. Mice received 5x10^5^ naïve CellTrace Violet-coated OT-I and PbT-I cells one day before injection of 5.2-8x10^4^ HsOVA HKS, and numbers of divided OT-I and PbT-I cells were quantified in the spleen 4 days later. Data were pooled from two independent experiments, log-transformed and analysed using unpaired Student’s T-tests. F. Distribution of memory T cells of the indicated specificities in the liver and the spleen of mice immunised with HsOVA RAS. Data were pooled from two independent experiments, log-transformed and analysed using two-way ANOVA and Tukey’s multiple comparisons test. The statistical analysis performed on liver data compared numbers of TRM cells. (TIF [file ppat.1012731.s005.tif]

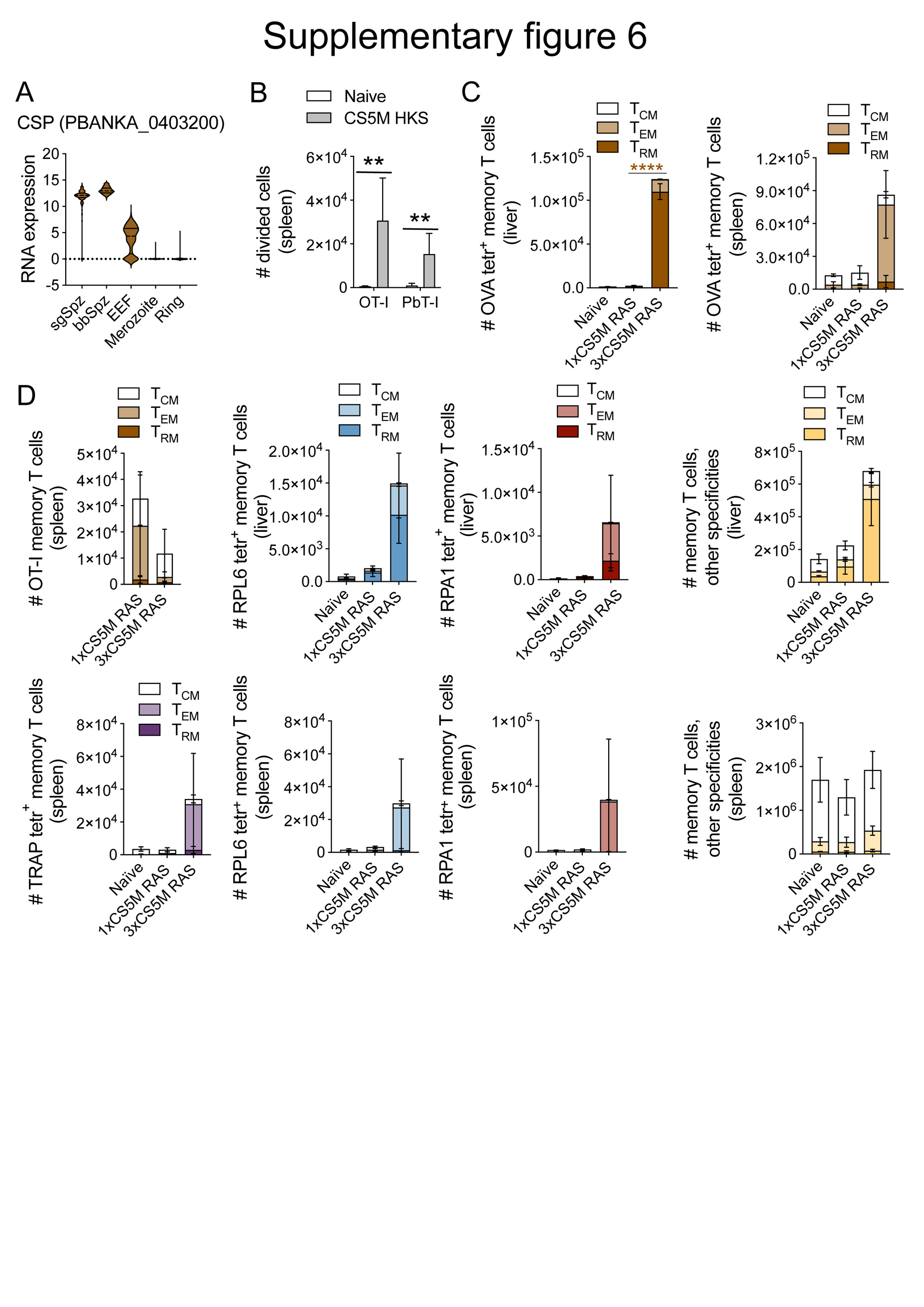

Supplement: S6 Fig — A. Expression of CSP across different life stages of the parasite, as per the Malaria Cell Atlas [33]. B. OT-I and PbT-I cell expansion after CS5M HKS injection. Mice received 5x10^5^ naïve CellTrace Violet-coated OT-I and PbT-I cells one day before injection of 4.2-4.5x10^4^ CS5M HKS, and numbers of divided OT-I and PbT-I cells were quantified in the spleen 6 days later. Data were pooled from two independent experiments, log-transformed and analysed using unpaired Student’s T tests. C-D. Related to Fig 5I and 5J. Distribution of OVA-specific endogenous memory T cells (C) and other specificities as indicated (D) in the liver and the spleen. Data were pooled from two independent experiments (except for endogenous OVA and RPA1, which were measured in one experiment), log-transformed and analysed using one-way ANOVA and Tukey’s multiple comparisons test. (TIF) [file ppat.1012731.s006.tif]

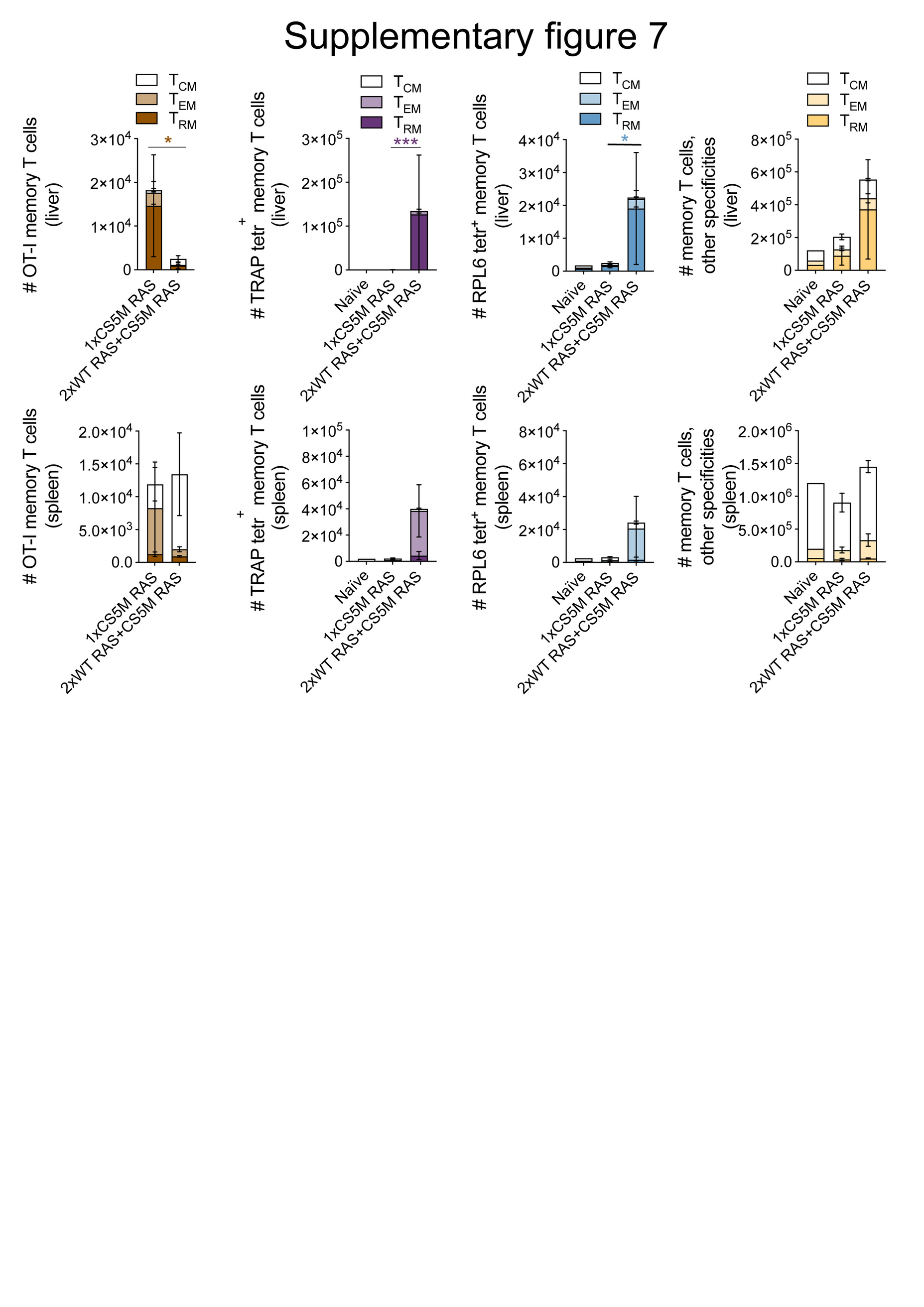

Supplement: S7 Fig — Memory CD8+ T cells were enumerated in the liver and the spleen of mice vaccinated with 2 doses of 5x10^3^ and 10x103 WT RAS 4 days apart, then transferred with 50x103 naïve OT-I cells and given a final dose of 5.1x10^3^ CS5M RAS 8 days later, or control mice receiving OT-I cells and one dose of 5.1x10^3^ CS5M RAS. Mice were euthanised on day 62 after the last sporozoite injection. Data were generated in one experiment, log-transformed and analysed using one-way ANOVA and Tukey’s multiple comparisons test. The statistical analysis performed on liver data compared numbers of TRM cells. (TIF) [file ppat.1012731.s007.tif]

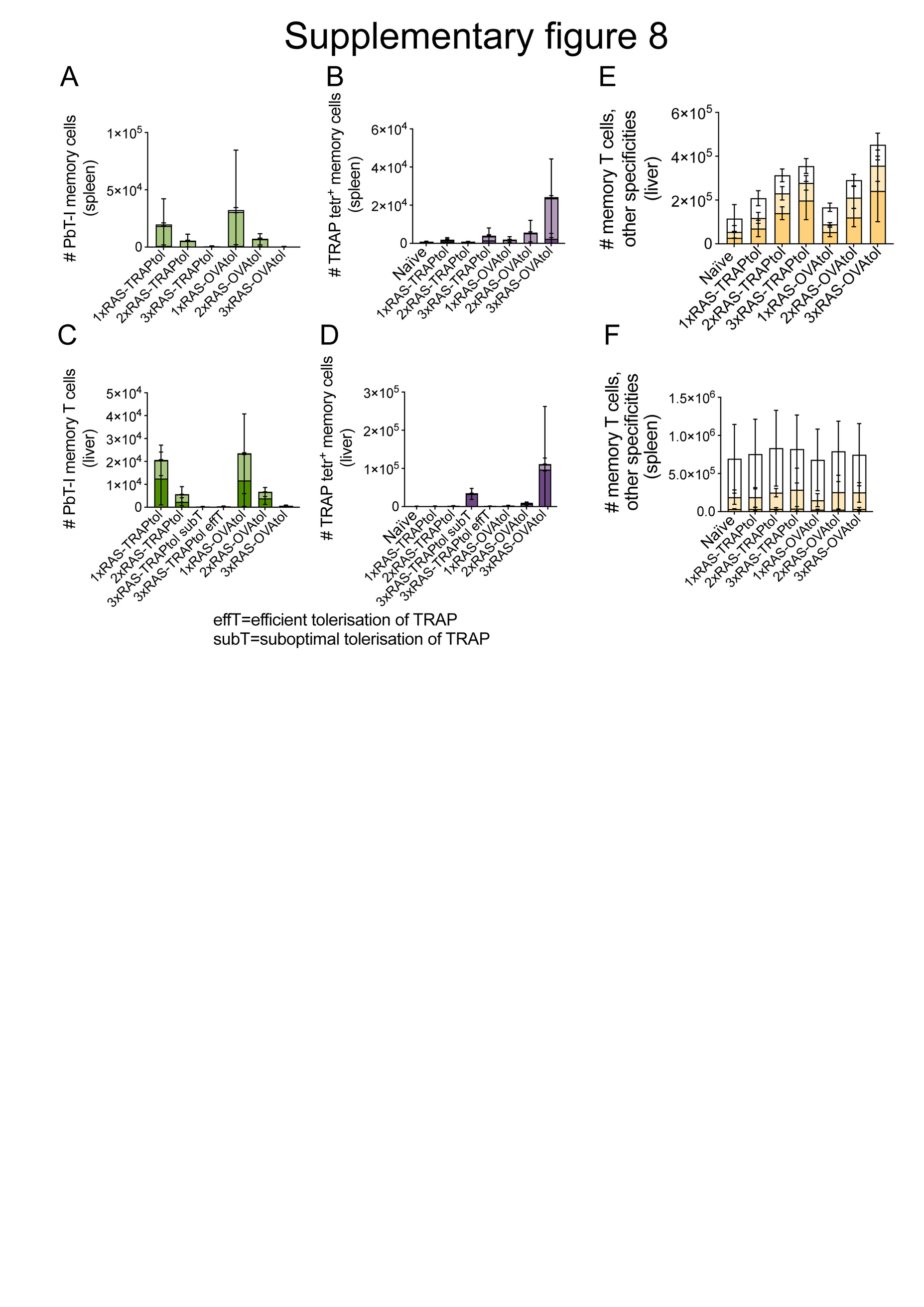

Supplement: S8 Fig — Distribution of TCR transgenic and endogenous memory T cells in RAS-vaccinated mice that were efficiently or suboptimally tolerised for PbTRAP130–138. A. Memory PbT-I cells in the spleen. B. TRAP-specific memory CD8+ T cells in the spleen. C-D. Numbers of PbT-I (C) and TRAP-specific (D) memory cells in the liver as in Fig 5K and 5L, but mice in the 3xRAS group in which TRAP tolerisation worked efficiently (effT) or suboptimally (subT) were separated into different columns. E. Endogenous memory CD8+ T cells of undefined specificities (non-TRAP) in the liver. F. Endogenous memory CD8+ T cells of undefined specificities (non-TRAP) in the spleen. (TIF) [file ppat.1012731.s008.tif]
