# Supplementary material for: Long lived liver-resident memory T cells of biased specificities for abundant sporozoite antigens drive malaria protection by radiation-attenuated sporozoite vaccination
Source: PLoS Pathog. 2025 May 27;21(5):e1012731. doi: 10.1371/journal.ppat.1012731 (PMC12143544; doi:10.1371/journal.ppat.1012731)
Supplement: Attachment — Submitted filename: PLOS Pathogen review report.docx [file ppat.1012731.s010.docx]

Part-I: The study by Menezes et al. finds a very interesting observation the critical role of Trm in ensuring the protection to Plasmodium liver-stage infection. It is a novel study clearly showing that the depletion of Trm abrogates the protection to sporozoite challenge (1).

Interestingly, it shows that the CD8 Trm are largely restricted to spz or early liver-stage Ags, TRAP and CSP (2).

They have also shown that there is inhibition of T cell responses to other antigen expressed at later stages limiting the potential of RAS vaccination (3).

Part-II: 1) Although understandable, it is intriguing to know that the Trm/Tem against CSP/TRP inhibit the T cell responses to later liver-stage antigens. The explanations provided by the authors sound reasonable. However, it would be interesting to know the T cell repertoire upon Infectious spz challenge following RAS vaccination. Since γ-spz upon invading the hepatocytes is known to express restricted antigens, the scenario would change when inf-spz is inoculated in form of challenge. It is expected that level and expression profiles of antigens of later liver-stage would be enhanced despite the infected hepatocytes under attack by the CD8 Trm/Tem. This would lead to expansion of T cells against late stage antigens.

2) Authors claim that repeated RAS improves the longevity of CD8 Trm. This should be reflected in the longevity of protection to spz challenge after 200+ days post vaccination. However, the protective potential has been tested only after 30 days post vaccination. It would add value to their findings if the authors do a kinetic study of protection beyond day 200 post vaccination.

Part-III: It would be helpful if the schematics of flow-analysis could be inserted (minimally) in the main figures.

Confidential Comments to Editor: If the suggested exp. # 1 can't be carried out by the authors, it is okay to accept the paper.
