# Supplementary material for: Long lived liver-resident memory T cells of biased specificities for abundant sporozoite antigens drive malaria protection by radiation-attenuated sporozoite vaccination
Source: PLoS Pathog. 2025 May 27;21(5):e1012731. doi: 10.1371/journal.ppat.1012731 (PMC12143544; doi:10.1371/journal.ppat.1012731)
Supplement: Attachment — Submitted filename: deMenezes_Response to reviewers.docx [file ppat.1012731.s012.docx]

**Response to reviewers**

*We sincerely thank the three reviewers for their constructive feedback on our manuscript. We have carefully considered and thoroughly addressed all their concerns, which has significantly strengthened our manuscript.*

**Reviewers' Comments:**

**Part I – Summary**

Please use this section to discuss strengths/weaknesses of study, novelty/significance, general execution and scholarship.

Reviewer #1: This paper reports excellent descriptive and mechanistic immunology findings regarding immunization of mice with early arresting, replication deficient radiation attenuated sporozoites (RAS) of Plasmodium berghei (Pb). If the importance of these murine malaria studies, is to improve the development of human PfSPZ vaccines, the authors to my mind, have not communicated how this would be done. The first step, which the authors have not done optimally, is to put their findings in perspective with clinical findings with injectable, early arresting replication deficient radiation-attenuated and genetically-attenuated Plasmodium falciparum sporozoite vaccines (PfSPZ Vaccine and PfSPZ-GA1) and late arresting, replication competent chemo-attenuated (PfSPZ-CVac) and genetically-attenuated (PfSPZ-GA2 and PfSPZ-LARC2 Vaccine) PfSPZ vaccines.

*We thank this reviewer for their thorough review of our manuscript and their effort in suggesting specific references. When writing this manuscript, we did not initially focus on the point of improving human vaccination, but this has been a concern all three reviewers have directly or indirectly raised. In response, we have revised the manuscript to incorporate more references to current clinical data, as suggested by this reviewer. Additionally, we have included new data that provides a mechanistic explanation for the improved efficacy of higher doses of RAS. Detailed responses to specific points are provided below.*

The authors conclude in the Abstract, “These findings provide novel insights into the mechanisms governing malaria immunity induced by attenuated sporozoite vaccination and highlight the susceptibility of this vaccine to limitations imposed by strain-specific immunity associated with the abundant, yet highly variable sporozoite antigens CSP and TRAP.”

At the end of the Introduction they state, “Our findings reveal a progressive skewing of the liver TRM response towards sporozoite antigen specificities, which undergo robust expansion and become major mediators of protection against live sporozoite challenge. Surprisingly, this immunodominance is established even for T cells targeting the sporozoite antigen TRAP, despite the modest intrinsic protective capacity of these cells. Additionally, repeated immunisations significantly extend the half-life of the parasite-specific TRM cells generated while suppressing naïve T cell responses to any parasite antigen. These elements limit the breadth of the ensuing memory response by largely constraining the induced liver TRM cell pool to cells specific for previously encountered sporozoite antigens.”

At the end of the Results they state: “Together, this study shows that repeated RAS vaccination enhances the development of long-lived liver-resident memory CD8+ T cells of biased specificity for abundant sporozoite antigens, some of them highly protective, but fails to fully exploit the protective potential of T cells specific for less abundant or later antigens.”

At the end of the Discussion, they emphasize the fact that RAS immunization does not induce immune responses against late liver stage antigens, which may be important for protective immunity.

From my perspective, the findings in this paper provide us with a more granular understanding of what we have known for years. Much of what they describe is already known from clinical trials, and in some cases, the limitations they highlight (e.g. strain specificity) appear to have been overcome. The authors need to describe and reference these findings, which they for the most part only describe broadly. They need to put their findings in the context of what we already know and describe how what they find can enhance clinical vaccinology with PfSPZ.

*The primary aim of the work we present here is to use mouse studies to provide detailed mechanistic insights into phenomena that cannot be studied in humans with current tools. Today, these mechanisms can only be demonstrated in mice for us to understand human data.*

*Although human studies, and the animal studies that preceded them, have provided substantial insight into the immunological mechanisms at play during RAS vaccination, we here provide a number of novel concepts: 1) the progressive increase in liver Trm cells upon repeated RAS vaccination, 2) their enhanced life span, 3) the bias for abundant sporozoite vs less abundant or later antigens and 4) the impairment of naïve T cell responses, 5) the reliance on sporozoite specific cells for protection are all novel concepts. We therefore disagree in that the science we present here has been known for years.*

*Only the recent identification of liver Trm cells, and of some Plasmodium antigens, such as RPL6, and the development of tools for their study, have enabled us to undertake the comparative study of T cell specificities elicited by RAS vaccination that we present in this manuscript. Most of our findings were quite surprising to us when first obtained. For instance, the strong inhibition of naïve CD8 T cell responses (even to CSP) in mice previously vaccinated with RAS was quite unexpected, and certainly not easily inferable beforehand. Knowledge of the mechanistic immunological basis for improved vaccination is essential for further development of the field, and the rational improvement of current vaccines.*

*We believe the limitations of whole sporozoite vaccination have not been fully addressed. Achieving substantial heterologous protection requires large doses of RAS, and efficacy in malaria endemic regions is not yet optimal. Understanding the immunological mechanisms underlying these shortcomings is not detrimental, but essential to refine vaccination strategies and avert future failures. Clinical trials, though valuable for evaluating outcomes, are inherently unsuitable to study these mechanisms – their results must be interpreted through fundamental immunological principles. Nevertheless, we are thankful for the reviewer’s feedback and, in line with the main objectives of this manuscript, have incorporated new data elucidating why higher RAS doses enhance protection (see our answers to specific points below).*

1. The authors demonstrate that repeated RAS immunization primarily induces CD8 T cell responses against two antigens, CSP and TRAP. These proteins, expressed in sporozoites and in liver stages, were discovered more than 30-40 years ago, and because they are the primary two antigens for RAS, they have been the immunogens in more than 95% of all human pre-erythrocytic stage malaria vaccines. The findings need to be put in perspective with these facts.

*We have extended the introduction to include more information about these antigens (starting at line 113 in the change-tracked version and 112 in the clean version of the revised manuscript).*

2. Repeated immunization leads to increasing parasite specific tissue resident T cells but not peripheral T cells. This is consistent with the multiple clinical trials that show that T cell responses in PBMCs decrease after priming immunization with PfSPZ Vaccine. This should be described and referred to (see Jongo JCI, 2024 for an example and references).

*Thank you. We had cited some papers in relation to this point (Epstein et al, Lyke et al, Jongo et al, line 511 in the original submission) but have included the additional reference and made a more specific mention of it, line 511 (change-tracked manuscript)/505 (clean manuscript).*

3. The hypothesis that late arresting replication competent pre-erythrocytic stage vaccines will be more effective/potent than early arresting. This has been obvious since it was shown that 3 doses of 50,000 PfSPZ of PfSPZ-CVac at 4-week intervals gave 100% protection against homologous CHMI at 10 weeks after last dose of vaccine and 3 doses of 12,800 PfSPZ gave 67% protection at 10 weeks after last dose (Mordmüller, Nature, 2017) whereas 3 doses of 900,000 PfSPZ gave 64% protection at 19 weeks (Lyke, PNAS, 2017). This was further delineated when 3 doses of 900,000 PfSPZ on days 1, 8, and 29 gave 79% protection against heterologous CHMI at 9-10 weeks after last vaccine dose (Mordmüller NPJ Vaccines, 2022) and 110,000 PfSPZ of PfSPZ-CVac gave 77% protection against heterologous CHMI at 12 weeks after last dose (Sulyok, Nature Communications, 2022); an 8-fold lower dose gave similar protection and 3 doses of 200,000 PfSPZ gave 100% protection against heterologous CHMI at 12 weeks (Mwakingwe-Omari, Nature, 2021), a level of protection against heterologous CHMI unmatched by any other malaria vaccine. This has now been dramatically shown by mosquito bite immunization with early vs late arresting genetically attenuated PfSPZ in humans (Lamers, NEJM, 2024), and in mice (Goswami, EMBO Mol Med, 2024, and JCI Insight 2020). Thus, it has been well known from clinical trials (and murine studies) that early arresting, replication deficient PfSPZ are much less potent than late arresting, replication competent PfSPZ (and PySPZ). It is not known if this is due to the 10,000 to 50,000-fold increase in parasite mass, the exposure of the immune system to thousands of proteins not expressed by early arresting parasites, or the skewing of the immune responses to CSP and TRAP described in this paper with RAS. This needs to be described and referenced.

*We thank the reviewer for the detailed information. We indeed referred to these points in our original submission by citing the Harty paper (ref 33 in original submission) that originally described this phenomenon in mice in 2011, by indicating that “Vaccination with genetically modified parasites engineered to interrupt their intrahepatic development at a late stage after hepatocyte invasion induces more protective T cell responses than early arresting parasites, which express a more limited antigen breadth [33]“ and “late antigens with prolonged expression patterns provide a longer window of opportunity for T cells to find and eliminate parasites before they progress to the blood”. We also explained in the discussion that “Administration of high doses of RAS, however, may aid initial expansion of protective T cells specific for less abundant sporozoite antigens and increase cross-strain immunity”, citing Lyke, PNAS, 2017 (ref 25). Our manuscript focuses on RAS and does not compare early or late arresting parasites. However, we are happy to elaborate more on this point and have now included the extra references proposed to make this point clearer. In line 555(change-tracked)/549(clean version) onwards, we added additional references to human clinical trials with GAP and PfSPZ-CVac (Lamers, Roestenberg, Mordmuller, Mwakingwe-Omari). Starting in line 756/744, we include a new paragraph discussing these results.*

*Additionally, we now provide evidence that supports superior immunogenicity of higher doses of RAS for less abundant sporozoite or later antigens: we can compare numbers of TRM cells specific for different antigens generated in mice injected once with a low dose of RAS (10,000), or with a higher dose (50000 RAS). The result is that 50k RAS induces more Trm of each specificity, resulting in more later stage-specific cells (RPL6, RPA1). Indeed, the ratio of numbers of liver Trm cells of each specificity in 1x10kRAS or 1x50kRAS vs Naïve mice clearly shows larger increases in the 50k group, and that later stage antigens to expand comparably more than early ones. As T cells specific for these antigens activated upon the first dose do continue expanding over subsequent doses, we reason that the increased numbers of these cells generated with higher immunisation doses lead to higher memory T cell numbers overall targeting these late stage antigens, hence improving overall protection. These results provide mechanistic explanation for the question posed by the reviewer: sporozoite bias is still likely to occur when GAP or live parasites under drug cover are used for immunisation, as abundant sporozoite antigens are still present, but the initial larger expansion of late liver stage T cell specificities likely allows further expansion upon subsequent doses and better protection.*

*We have included this new comparison in figure 2I-K.*

4. Furthermore, it has been shown in human studies, which were not referred to on these particular points, that altering the immunization regimen with PfSPZ Vaccine (RAS) (number of doses, interval between doses, and strength of each dose) can increase the protection against heterologous CHMI (Mordmüller, NPJ Vaccines, 2022) and increase protection against field transmitted malaria (Diawara, Lancet ID, 2024). Is this because the limitations of RAS immunization the authors point out, lack of induction of sub-dominant epitopes, can be overcome? These findings need to be discussed.

*As per our answer above, we now show that increasing doses increases generation of Trm cells specific for later/less abundant epitopes, and this likely result in improved efficacy. We also discuss clinical trial results under the light of the novel findings presented in this manuscript.*

Reviewer #2: This study in the Pb/C57Bl6 malaria model shows the skewed antigenic breadth induced by subsequent Radiation Attenuated Sporozoite (RAS) immunizations. Established protective well known target antigens are dominated by TRAP and CSP, highly expressed in sporozoites. De data provide insight to a phenomenon that is well known.
The studies are well designed and conducted and the paper reads well although the elaborate Discussion can to be substantially condensed without repeats from the Results section.

*We have now simplified parts of the discussion trying to avoid repeating the results section.*

Reviewer #3: The study by Menezes et al. finds a very interesting observation the critical role of Trm in ensuring the protection to Plasmodium liver-stage infection. It is a novel study clearly showing that the depletion of Trm abrogates the protection to sporozoite challenge (1). Interestingly, it shows that the CD8 Trm are largely restricted to spz or early liver-stage Ags, TRAP and CSP (2). They have also shown that there is inhibition of T cell responses to other antigen expressed at later stages limiting the potential of RAS vaccination (3).

**Part II – Major Issues: Key Experiments Required for Acceptance**
Please use this section to detail the key new experiments or modifications of existing experiments that should be absolutely required to validate study conclusions.

Generally, there should be no more than 3 such required experiments or major modifications for a "Major Revision" recommendation. If more than 3 experiments are necessary to validate the study conclusions, then you are encouraged to recommend "Reject".

Reviewer #1: (No Response)

Reviewer #2: 1. The effector cells and mechanisms of protection are specific to this particular malaria model. I wonder how much the skewed antigenic breadth is determined by the H2-genotype and therefore rather a finding for a specific model than a general phenomenon. Is host genotype not a major determinant of responsiveness? Would the authors expect a similar outcome in outbred mice? Please discuss.

*Host genotype is certainly a major determinant of responsiveness. For example, as mentioned in our manuscript, B6 mice cannot develop CD8 T cell responses to P. berghei CSP, but BALB/c CD8 T cells do respond to P. yoelii CSP. We used different models (e.g. the CS5M parasites) to overcome the host limitation, by introducing a B6 epitope in CSP, and hence be able to measure T cell responses to this protein in this model. We obtained similar results as with TRAP, validating our hypothesis that sporozoite antigens are generally favoured in RAS vaccination. In line with this concept, we would expect that RAS-vaccinated outbred mice (and humans) capable of developing CD8 T cell responses to abundant sporozoite antigens will be susceptible to the specificity bias we describe in our manuscript. We have included a comment on this in lines 583-586 (change-tracked manuscript version) / 577-580 (clean version).*

2. This dominant role for protection is striking and in fact not good news for non-TRAP/ CSP proteins as potential vaccine targets using whole sporozoite vaccines. However, there is strong evidence that late arrest of developing parasites favors more potent and protective immunity. This in facts moves away from the key importance of spz or early liver stage antigens as preferred immunogens. Can the authors comment and provide an outline to address this point for further studies possibly with liver-stage drugs or late-arresting gene deletion mutants.

*This comment is in line with those from reviewer 1. To address it, we now provide new data on the frequency of T cell specificities generated when using larger doses of RAS (figure 2I-K). We have also extended our discussion to elaborate on this point (line 555; paragraph starting on line 756 in change-tracked manuscript/ line 549 and paragraph starting on line 744 in clean version).*

3. Studies from decades ago (nineties) with a number of infectious agents including Plasmodium have shown to induce skewing of the specificity upon subsequent immunizations. They were explained by clonal imprinting. Could this phenomenon play a role in this study?

*We think this is what we see here. A competition between T cell clones whereby some specificities are more favoured than others initially, likely based on the abundance/ expression window of an antigen, whereby this dominance is maintained over subsequent exposures to antigen. We discuss this in the last paragraph (starting on line 777 of the change-tracked version/765 of the clean version) in the context of the new data included in the manuscript, and provide an additional reference on this point (Lin et al, ref 117).*

4. Fig 4: The data show that TRAP tolerance reduces both the induction of positive TRAP-tetramer cells and protective efficacy but is the total RAS specific T cell reservoir similar i.e. combined response to other antigens increased?. Did the numbers of CD8 cells with RPA1, RPL6 and other specificities remain similar to fig 2? They may have increased? This would provide insight on their potential role in protection.

*Note that removal of TRAP-specific CD8 T cells removes protection. This implies that, in the absence of these cells, non-TRAP specific cells are unable to provide substantial protection.*

*In SF8 we show that total numbers of endogenous, non-TRAP specific liver TRM cells in the spleen and the liver were equivalent in TRAP- and OVA-tolerised mice – only slightly lower in the former group, indicating that other specificities do not compensate for the lack of TRAP-specific cells.*

5. A general limitation of the murine malaria models is the relative short (48hr) of Pb versus 7 day liver-stage period of human malaria as pointed out by the authors. Spz antigens become increasingly dominant upon subsequent immunizations with RAS but limit the generation of T-cells targeting (late) liver stage. What do the authors see as the way forward for strategy of pre-erythrocytic vaccine development?

*This is in line with the comments by reviewer 1. We now provide additional data on immunisation with 50kRAS suggesting that a larger initial dose may increase numbers of late stage specificities (figure 2I-K).*

6. There is also a quantitative aspect as immunogenicity also depends on dose of expressed targets over time during Pb liver stage. Could the immunogenicity and dominance of TRAP and CSP be explained by their quantitative abundance?

*We certainly think this is the case, and we discuss this point throughout the manuscript. We indeed refer to TRAP and CSP as abundant sporozoite antigens, whereas other antigens (including RPL6 and RPA1) are referred to as “less abundant or later antigens”. For example, RPL6 is expressed during the sporozoite stage, as evidenced by PbT-I cell responses to heat-killed sporozoites, but its immunogenicity at this stage is markedly inferior to that of dominant antigens like CSP or TRAP. However, conclusively demonstrating antigen-specific immunogenicity hierarchies based on abundance is methodologically challenging given the complex nature of immunity to liver stage malaria. Parasites engineered to express different amounts of the same antigen during the same temporal window would be needed to formally prove this point. Our findings leverage dose-response dynamics and T cell specificity patterns to infer antigen abundance and availability.*

Reviewer #3: 1) Although understandable, it is intriguing to know that the Trm/Tem against CSP/TRP inhibit the T cell responses to later liver-stage antigens. The explanations provided by the authors sound reasonable. However, it would be interesting to know the T cell repertoire upon Infectious spz challenge following RAS vaccination. Since γ-spz upon invading the hepatocytes is known to express restricted antigens, the scenario would change when inf-spz is inoculated in form of challenge. It is expected that level and expression profiles of antigens of later liver-stage would be enhanced despite the infected hepatocytes under attack by the CD8 Trm/Tem. This would lead to expansion of T cells against late stage antigens.

*For this experiment to be meaningful, mice challenged with live sporozoites would need to be protected against this challenge. This would be achieved through repeated RAS immunisation, inherently introducing the T cell repertoire bias. As live sporozoites contain large amounts of abundant sporozoite antigens, and T cells targeting these antigens will respond and exert efficient protection, we reason that this scenario would leave little opportunity for alternative specificities to expand. We now include new data that provides an alternative way to induce more of these T cells specific for less abundant antigens, namely to immunise using larger initial doses, which leads to stronger early generation of T cells of these specificities (figure 2I-K).*

2) Authors claim that repeated RAS improves the longevity of CD8 Trm. This should be reflected in the longevity of protection to spz challenge after 200+ days post vaccination. However, the protective potential has been tested only after 30 days post vaccination. It would add value to their findings if the authors do a kinetic study of protection beyond day 200 post vaccination.

*The Nganou-Makamdop paper (ref 6) already tested protection in 3xPbRAS B6 mice for up to 9 months. This regime provided 100% protection and this was indeed better than immunisation using live sporozoites under chemoprophylaxis, which only resulted in 50% protection at 9 months. We now make a specific mention of these results in the discussion (lines 713-719 of the change-tracked manuscript/701-707 of the clean version)*

**Part III – Minor Issues: Editorial and Data Presentation Modifications**
Please use this section for editorial suggestions as well as relatively minor modifications of existing data that would enhance clarity.

Reviewer #1: 1. Line 77, references 3-5. None of these references show long-lived immunity that is referred to. Use Hoffman, JID, 2002, Ishizuka Nat Med 2016, and most importantly Diawara Lancet ID, 2024.

*The purpose of the statement the reviewer is referring to was to simply highlight the fact that RAS protects across species, in mice/NHP/humans. We realise that we should either not have alluded to long lived protection, or have provided additional references to substantiate persistent protection. Since this is a valuable feature of RAS, we have decided for the latter and incorporated the references the reviewer suggested, plus Nganou-Makamdop 2012 to substantiate long lived protection also in mice (lines 81-83 in the change-tracked manuscript version/79-81 in the clean version). We thank the reviewer for suggesting these references.*

2. Line 128. “Despite the modest intrinsic protective capacity…” In regard to protective immunity induced by TRAP/SSP2, should reference Khusmith, Science 1991 (immunization alone gave 50% protection, immunization alone with CSP gave 67% protection and the combination of CSP and SSP2 gave 100% protection) and Khusmith, Infection and Immunity, 1994 (T cell clone) for original demonstration of protection induced by TRAP/SSP2.

*These results align with our current and prior research: we demonstrate in this manuscript that TRAP specific cells can provide efficient protection against infection. However, in the statement mentioned by the reviewer we are referring to a per-cell basis. Our prior work has shown that in mice harbouring similar number of TRAP- or RPL6-specific liver Trm cells, the latter are better protected, indicating that the former cells are comparatively less protective. Also, a major element that was not clearly mentioned in this sentence but is relevant is the fact that TRAP-specific CD8 T cells are very infrequent in B6 mice. We have now clarified this point and added the relevant references (lines 146-150, change-tracked version/143-147, clean version).*

*We have also extended the discussion on this point (line 686 onwards, change-tracked version/674 clean version): we here show that even lower numbers of CSP-OVA specific cells are needed for potent protection than RPL6, reinforcing the concept that TRAP responses are comparably less efficient that those specific for other antigens. Importantly, Khusmith targeted P. yoelii TRAP in BALB/c mice, and P. yoelii and P. berghei differ in the features of liver stage development (as we discuss in the manuscript) and potentially in the T_RM_ cell specificities required for protection.*

3. Line 210-. It would be useful to the reader to show a figure demonstrating the timeline of expression of CSP, TRAP, RPL6, and RPA1 in livers of mice infected with RAS and wild type Pb.

*With current data, it is difficult to represent the exact expression pattern of Plasmodium antigens during different time points of liver stage, and hence a figure on this point may be inaccurate. We preferred to use data from the malaria cell atlas to provide an indication of the RNA expression at different life stages – included within supplementary figures throughout the paper.*

4. Line 481: In humans, protection was maintained in RAS-vaccinated individuals a year after vaccination, a time when numbers of circulating T cells and antibodies had declined to background levels [13]. Should refer to and reference, Diawara, Lancet ID 2024. Protection with PfSPZ Vaccine lasted two years against heterogeneous parasites after only 3 doses of PfSPZ Vaccine administered on days 1, 8, and 29.

*In the sentence referred to by the reviewer, we did not intend to highlight that RAS can induce long term protection, but that the protection induced by RAS was maintained at a time when circulating cells were undetectable, suggesting that local immunity in the liver (but not circulating cells or antibodies) was protective. We have now specified this more clearly (line 511-512, change-tracked version/505-506, clean version).*

5. Line 489: Note that the best protection in the field (Diawara, Lancet ID 2024) was with 3 doses on days 1, 8, and 29, a contracted, minimal dose regimen. How might this be enhancing protection based on your data?

*We think the Diawara immunisation schedule is largely equivalent to the model we are showing here, and we now discuss this (line 762-763, change-tracked version/750-551, clean version). We have not attempted to delay the third dose, but it is conceivable that a similar result would be achieved as with three weekly doses used here (as this latter regime already provides highly efficient protection). Once mice/humans have received two doses of RAS a week apart, and generated good numbers of T cells with potentially enhanced longevity, it is conceivable that delaying the third dose by a week would not have a major effect.*

6. Line 515: It appears in people that giving two priming doses at 1 week interval and a boosting dose 3 weeks later, in some respects overcomes the limitation of antigen specificity described here. Need to discuss how this double priming may alter the responses they describe.

*We now incorporate the 1x50k RAS data to help address this point (figure 2I-K) – by providing a larger dose initially, more of the cells specific for late stage antigens are generated. We show that these cells continue expanding after every dose of RAS, hence resulting in larger numbers of TRM cells specific for less abundant or later liver stage antigens.*

7. Line 525 and 526. Reference 33 is outdated. Need to reference and describe the recent NEJM article demonstrating the dramatic difference in protective efficacy of GA1 (early arresting replication deficient) and GA2 (late arresting replication competent) after mosquito bite immunization; a point also made in Goswami (JCI Insight 2020 and EMBO Mol Med 2024).

*Ref 33 in the original submission is the original work that compared CD8 T cell responses in RAS vs GAP in mice, and the later papers mentioned build up on it and should be interpreted under the mechanistic frameset provided by this foundational paper by the Harty group. We now cite all these newer papers in the discussion. The Goswami papers did not study T cell responses or compared them to RAS, but we now reference them when discussing the antigenic breadth expressed by different types of parasites (line 555, change-tracked version/549, clean version).*

8. Line 537. Recognizing that Pb may be different than Py and Pf, it would be useful to the reader to reference the original articles on CD8 dependence of TRAP/SSP2 induced protection (Khusmith, Science, 1991), and the articles that showed that immunization of humans with Pf RAS induced CD8+ CTL against SSP2/TRAP (Wizel, JEM and JI, 1995, Doolan, Immunity 1997).

*Thank you; we have added these references.*

Reviewer #2: 1. 488”: Additionally, protection increases with successive RAS injections, mirroring observations in humans [9]. This mouse model hence presents strong analogies with humans. I don’t think that the parallel observation in mice and humans is sufficient for this claim. Moreover it is not relevant for the findings in this murine malaria model.

*Our statement is not only based on this single statement about increasing protection. We mentioned several other features (dependence on CD8 T cells, likely dependence on Trm cells) that makes it very similar to humans, to our view*.

2. Abstract (43): “to all parasite antigens in previously vaccinated mice”. Unclear please rephrase

*We have modified this for better clarity; “to parasite antigens, previously encountered or not”.*

Reviewer #3: It would be helpful if the schematics of flow-analysis could be inserted (minimally) in the main figures.

*We decided to include these as supplementary figures, as the main figures are quite full. SF1 has the general gating schematic used for all other figures, and we have included additional representative FACS plots when relevant (SF2C, SF3A).*
